# Addressing persistent evidence gaps in cardiovascular sex differences research – the potential of clinical care data

**DOI:** 10.3389/fgwh.2022.1006425

**Published:** 2023-01-20

**Authors:** Sophie H. Bots, N. Charlotte Onland-Moret, Hester M. den Ruijter

**Affiliations:** ^1^ Division of Pharmacoepidemiology and Clinical Pharmacology, Utrecht Institute for Pharmaceutical Sciences, Utrecht University, Utrecht, Netherlands; ^2^ Laboratory for Experimental Cardiology, University Medical Center Utrecht, Utrecht University, Utrecht, Netherlands; ^3^ Julius Center for Health Sciences and Primary Care, University Medical Center Utrecht, Utrecht University, Utrecht, Netherlands

**Keywords:** real world evidence (RWE), sex differences, cardiovascular disease, electronic health records (EHR), women, epidemiology

## Abstract

Women have historically been underrepresented in cardiovascular clinical trials, resulting in a lack of sex-specific data. This is especially problematic in two situations, namely those where diseases manifest differently in women and men and those where biological differences between the sexes might affect the efficacy and/or safety of medication. There is therefore a pressing need for datasets with proper representation of women to address questions related to these situations. Clinical care data could fit this bill nicely because of their unique broad scope across both patient groups and clinical measures. This perspective piece presents the potential of clinical care data in sex differences research and discusses current challenges clinical care data-based research faces. It also suggests strategies to reduce the effect of these limitations, and explores whether clinical care data alone will be sufficient to close evidence gaps or whether a more comprehensive approach is needed.

## Introduction

Women have historically been underrepresented in cardiovascular trials, leading to scarcity of sex-specific data (1). This has resulted in a lack of knowledge on how to best diagnose and treat women with cardiovascular disease, regardless of whether they suffer from subtypes common in both sexes like heart failure and coronary heart disease (2, 3) or ones that occur more frequently in women such as Tako Tsubo cardiomyopathy and spontaneous coronary artery dissection (4, 5). In addition, current treatment guidelines often recommend the same treatment and dosages for women and men, despite known sex differences in drug metabolism (6) that relate to a 1.5–1.7 times higher risk of adverse drug reactions (ADRs) in women receiving cardiovascular medications (7). Importantly, the underrepresentation of women in cardiovascular trials has remained despite more than 20 years of effort to increase enrolment of women in these trials (8, 9). In addition, trials often do not present their safety and efficacy findings stratified by sex (10–12), further complicating research aiming to unravel sex-specific effects (13, 14). Hence, researchers need to look elsewhere for data to address these persisting evidence gaps. We argue that clinical care data could fit the bill nicely because of their unique broad scope across patient groups, time periods, and clinical measures. In this perspective piece, we will describe the potential of clinical care data for sex differences research and their inherent challenges, using ADRs as an example. We are aware that scarcity of data affects many facets of the sex differences research field, but believe the ADR case to be sufficiently representative to serve as an illustration of the potential of clinical care data. We will also discuss the future perspective and explore whether clinical care data alone will be sufficient to close existing evidence gaps or whether a more comprehensive approach is needed.

### Generating evidence where there is none: the potential of clinical care datasets

The main attraction of clinical care datasets is their potential to generate evidence in a relatively short time period, without the need for laborious and expensive data collection associated with clinical trials and cohort studies. In addition, they are a direct reflection of the patient population seen in daily care with proper representation of subgroups like women, the elderly, and those with multimorbidity. Work from our own group has illustrated how a clinical care dataset comprised of 110,000 cardiac outpatients (15) can address questions about cardiovascular disease prediction (16), risk assessment (17), and treatment (18, 19) in women. This illustrates that there are many areas where clinical care data can contribute, but for the sake of brevity we will focus on the case of ADRs. Attempts at elucidating sex-specific ADRs based on trial data have been hampered by the limited incidence of ADRs in combination with the underrepresentation of women in these trials (20). Pharmacovigilance data have hinted at sex-specific ADR combinations (21–23), such as a higher number of ACEI-related cough complaints in women (21). However, these studies are limited by their lack of information on sex-specific prescription rates. As a result, it is unclear whether the observed differences truly signal sex differences in ADR risk or are driven by differences in prescription rates between women and men. Two studies based on hospital data have supported the pharmacovigilance observations (24, 25), illustrating that clinical care date could be a valuable alternative source of information in this research area.

### Challenges in working with clinical care datasets

Clinical care data also come with their unique quirks that set them apart from data collected for research purposes (26–30). The most important difference is the reason behind data collection. Clinical care data is collected primarily for the purpose of delivering clinical care (28), meaning data collection is driven by medical need. This is inherently different from research datasets, where data is collected with the purpose of answering specific research questions.

Having medical need as the driver for data collection has various effects on the data, which can be divided into the *comprehensiveness* and the *quality* of the data (28). *Comprehensiveness* describes whether the dataset contains all relevant information about a single patient (28). Often clinical care datasets only capture a snapshot of the patient that contains the information clinically relevant at that time and thus do not tell a complete story. Information may remain out of reach, either because clinical care centres cannot collect information on patients that never visit or those that leave after being treated, or because one part of the system (hospitals) cannot access other parts (general practitioners) of the system, or because there is no medical reason to collect certain information. It may also be impossible to record certain information, for example due to time constraints or because equipment is not available, or because certain conditions do not yet exist as a designated diagnosis term or code (26, 29, 30). Existing beliefs about for example the likelihood that certain medication-ADR combinations occur in a sex-specific manner could also compromise *comprehensiveness* if either patients of one sex are more likely to report the ADR or physicians are more likely to specifically collect information on this ADR from one sex compared to the other due to this belief.

*Quality* is about how well the collected information reflects the truth about a single patient. Data entry in clinical care datasets is performed by healthcare professionals for healthcare professionals and is not subjected to extensive quality control. Entered information may be incorrect, inaccurate, incomplete, or incomprehensible (29, 30). These quality issues can arise both unintentionally (randomly) and systematically. Examples of the former are typos or something not being entered by accident. Examples of the latter are reimbursement coding systems that tend to mislabel certain conditions, diagnostic codes that change over time (29, 30), or the use of ambiguous abbreviations in free text summaries. Another important aspect of *quality* is whether the recorded information can be operationalised. For example, information recorded in free text format cannot be used without properly developed text retrieval methods (29). This is especially relevant for ADRs, as these are often only recorded within free text fields.

These considerations lie at the basis of deciding whether a clinical care dataset is *fit for purpose*, meaning that the information collected is of sufficient comprehensiveness and quality to answer the research question posed (26). Another concept that ties in with this is *validity*, which is about whether the size of random and systematic error interferes with drawing sound conclusions (27). In other words, if a dataset contains too many random errors such as typos or systematic errors such as mislabelling the condition of interest, it cannot reliably answer the research question. Taken together, these concepts offer researchers a crude guide for deciding whether or not to use clinical care data. However, this crude guide is not always sufficient to navigate the many nuances and grey areas surrounding the use of clinical care data. There are some published examples on how to deal with these kind of trade-offs (31–33) and a short guide on doing research with clinical care data (34), which we encourage readers to consult. However, the situation of sex differences research is unique in that in some areas the only information available comes from clinical care datasets. In this case, the opportunity to create new insights may easily outweigh potential limitations or drawbacks of a clinical data source. Nevertheless, there are four important questions to consider:

(1) Does the dataset cover the population of interest and collects the information required to answer the research question of interest?
(2) Are all members of the population of interest equally likely to be included and followed up in the dataset?
(3) Is missing data on relevant parameters not related to the exposure or outcome of interest?
(4) Can you reliably infer causality by correction for confounding (in the case of causal research)?

If the answer to any of these is “no”, the clinical data source cannot be used to answer the research question of interest. Sometimes the issue identified based on these questions can be mitigated to create the best possible alternative. For example, a lack of information on relevant outcome measures like ADRs (*question 1*) could be solved by operationalising the free text fields that contain this information. In cases where follow-up is not uniform across all individuals of the population of interest (*question 2*), passive follow-up through record linkage may be a solution. Several approaches to missing data (*question 3*) exist and these can address a variety of situations (35), except if missingness is truly related to the exposure or outcome of interest (not missing at random). Lastly, the choice of study design may reduce (unmeasured) confounding (*question 4*), which will be discussed in the next section.

Of course, choosing the best possible alternative will always be connected to inherent limitations. However, being hung up on those limitations and therefore abandoning all projects that cannot be performed under perfect conditions is unrealistic and undermines the potential advantages clinical care data can bring. In addition, these four questions can serve as a tool to separate clinically relevant questions that can be answered with clinical care data from those that cannot. This not only highlights the potential of clinical care datasets, but also identifies which clinically relevant questions still require a cohort study or clinical trial to be answered and thus should have priority on the research agenda.

Accessing clinical care data can also be challenging due to ethical issues surrounding patient privacy and safety. In the case of structured data, this can be mitigated by de-identifying potentially sensitive data. For example, records can be assigned a random record number instead of using patient numbers, dates of birth can be recoded into year of birth, and postal codes can be recoded into area codes. There are also algorithms available that can remove identifying information like names and dates from medical text (36). Another option is to adhere to a common data model, where the raw patient data remains with the local data owner but the structure and content (e.g., variable names, variable meanings) are standardised to a specific format also known by the researcher. The researcher can then prepare analysis scripts and let the local data owner run them, which means the researcher themselves never get direct access to the data and any sensitive information stored there (37, 38).

### The future perspective on clinical care data for sex differences research

There are two main developments that are important for the future of clinical care data-based research. The first are methodological advances that can mitigate the effect of data quality issues, such as more advanced text mining tools to extract information from free text fields. This is especially relevant for text that is not written in English, as currently available tools based on the English language may not function as well when applied to other languages (39). Moreover, clinical care data may require the development and application of novel study designs or analysis methods to deal with (unmeasured) confounding. The propensity score methods that originated in the counterfactual approach are one example (40), another is the use of self-controlled designs (41) or negative controls (42) in pharmacoepidemiologic studies of medication safety and effectiveness.

The second are improvements at the data-entry end, as better quality data input can potentially prevent many of the current issues from existing in the first place (28). In addition, such improvements can radically reduce the time needed for data cleaning and organising (‘data wrangling’) that currently takes up 60%–80% of time allocated for data science projects (43). There are many different ways to improve data entry, including stimulating healthcare professionals to adhere to data entry protocols, creating a data collection infrastructure that supports healthcare professionals in reliable data entry, or building tools that automatically turn messy speech or free text data into structured variables, among others (26, 28, 44). These should ideally be implemented with input from all parties involved, creating a feedback loop between researchers and healthcare professionals resulting in better quality data in the long term. This way, several of the current inherent limitations of clinical care data will slowly be filtered out, further pushing the balance towards the potential of these datasets to shed light on currently understudied topics in sex differences research.

### Closing existing evidence gaps, can clinical care data do it alone?

When faced with a daunting lack of sex-specific data, clinical care datasets may currently be the only resource one can turn to. Although clinical care data-based work generates momentum for sex differences research and pushes important sex-related questions into the limelight, its influence somehow does not reach daily clinical practice and the patients that may benefit.

In the current evidence-based medicine landscape, observational data only has a small role to play compared with clinical trials, which are considered the pinnacle of reliable evidence (45). This would mean that clinical care data can at most have a modest contribution in improving daily practice, even though they have so much potential. Clever study designs that incorporate the strengths of clinical care data into clinical trial settings, such as registry-based trials (46), have been suggested and applied to expand the sphere of influence clinical care data can exert. This innovative approach to study design could also be valuable for sex differences research, especially because there are questions that cannot be answered with clinical care data alone. For example, observational studies have hinted at benefits of sex-specific dosage but can never fully correct for the influences of confounding by indication and residual confounding (13, 47). Narrowing down the optimal dosing practice for each sex requires proper randomisation and dose-finding trials. In these situations, clinical care data can contribute by informing the field about which specific medications might merit additional trials, directing the limited resources available to the most relevant questions. Additionally, they could serve as the selection pool from which trial participants are included in these trials. However, this will only work in combination with other improvements that combat the underrepresentation of women and lack of sex-stratified reporting. Otherwise, the percentage of women participating in clinical trials may remain too low for meaningful sex-specific analyses, illustrated by the recent Low-Dose Colchicine 2 trial where only 15% of participants was female (48).

## Discussion

Summarising, questions regarding sex-specific medication dosage need a combination of clinical care and clinical trial data to be answered. However, clinical care data-based evidence should be given more credit in other fields of sex differences research, especially regarding ADRs. Clinical trials may be able to distinguish true ADR risk from any nocebo effects thanks to randomisation (49), but are otherwise not the best place to look for ADR data. With their highly specific study populations and relatively short follow-up times, trials are not powered to pick up on ADRs. Especially those that are rare or mainly occur in patient subgroups unlikely to meet the inclusion criteria. In addition, trials often actively exclude patients experiencing ADRs during the run-in period. Pharmacovigilance databases fill this gap, but lack information on prescription rates and patient-level characteristics. The latter in particular complicates the translation of pharmacovigilance findings to the clinic, because it is impossible to characterise the patient population the results pertain to. Clinical care data could have a big role to play here, especially when data entry becomes more standardised and of better quality.

However, caution is also advised because not all questions can be answered with clinical care data. It is important to realise that large amounts of data do not automatically guarantee proper quality data, especially because clinical care data are inherently different from data collected for research purposes. Researchers should always check whether a given clinical care dataset is *fit for purpose*. This perspective provides a four-question checklist that serves as the starting point for a *fitness for purpose* check, and we encourage researchers to use this checklist both when designing their own clinical care data-based studies and when reading clinical care data-based work from others.

To conclude, at the moment clinical care data offer many opportunities to generate interesting and relevant insights regarding existing evidence gaps, even though it cannot close them all without additional trial data. In addition, a *fitness for purpose* check is vital to ensure clinical care data-based research creates valid and reliable results. Nevertheless, as the quality of clinical care data and its research is only expected to grow in the future, the potential will further outshine the pitfalls, making it a valuable source for sex differences research.

## Data Availability

All datasets analyzed for this study are included in the manuscript.
